# Spigelian hernia, a case report

**DOI:** 10.1016/j.ijscr.2023.108785

**Published:** 2023-09-02

**Authors:** Safaa Hadi Abdulsattar Alshihmani

**Affiliations:** Al-kindy Teaching Hospital, Baghdad, Iraq

**Keywords:** Spigelian hernia, Hernioplasty, Linea Spigeli, Case report

## Abstract

**Introduction & importance:**

The first clinical presentation of a hernia developing along the Spigelian line had been reported by Klinkosch. The Belgian anatomist Adriaan van der Spieghel (Adrianus Spigelius) was the first to describe the semilunar line now known as the linea Spigeli in 1645. Spigelian hernias are rare and account for 1 % to 2 % of all abdominal wall hernias. Most of these hernia occurs in the lower abdomen where posterior sheath is deficient. The hernia ring is well defined defect in the transverse aponeurosis.

**Case presentation:**

A 60 year old female, presented with a palpable lump at the right lower quadrant of the abdomen since 7 month before her presentation.

**Clinical discussion:**

For the first time the swelling is small and painless then gradually increase in size and associated with dull aching pain. The swelling was reducible with a defect of size 4 × 4 cm palpable in right iliac fossa. There was a positive cough impulse. The swelling was non tender. Other hernial orifices were normal. No inguinal lymphadenopathy noted. Abdominal ultrasonography done revealed a defect in abdominal wall in right iliac fossa with reducible bowel content. Depending on basis of clinical and investigations, a diagnosis of Spigelian hernia was made. After preparation for surgery, exploration done. The defect measuring 4 cm in length was identified and anatomical repair was done with nylon- 0, by suturing medial border of internal oblique and transverse abdominus muscle to the lateral border of rectum abdominal wall followed by hernioplasty by mesh.

**Conclusion:**

Spigelian hernias are rare multifactorial disorder leading to defect in the transversus abdominis muscle in anterior abdominal wall. Spigelian hernias carry a significant risk of incarceration and strangulation of sac content. The management of spigelian hernias is almost always surgical which can be done in a traditional open fashion or laparoscopically.

## Highlights

• The first clinical presentation of a hernia developing along the Spigelian line had been reported by Klinkosch.
• Spigelian hernias are rare and account for 1 % to 2 % of all abdominal wall hernias.
• Most of these hernias occur in the lower abdomen where posterior sheath is deficient.
• Spigelian hernias carry a significant risk of incarceration and strangulation of sac content.
• The management of spigelian hernias is almost always surgical which can be done in a traditional open fashion or laparoscopically.

## 1 Introduction & importance

The first clinical picture of a hernia developing along the Spigelian line was described by Klinkosch [1]. The Belgian anatomist Adriaan van der Spieghel (Adrianus Spigelius) was the first to describe the crescent line known today with the line name Spigeli in 1645 [2]. They are rare, accounting for 1 to 2 % of all abdominal wall hernias [1].

The portion of the transversus abdominis aponeurosis between the lateral semilunar line and the lateral border of the medial rectus muscle is called the spigelian aponeurosis. Spigelian hernias are found between the layers of abdominal muscles along the semilunar line and are known as lateral ventral, interparietal, intermuscular, or intramural hernias. They retain a specific position along the semilunar line (spieghel), but are called “interstitial hernias” and should be distinguished from interstitial hernias [2].

Most of these hernias occur in the lower abdomen where posterior sheath is deficient. The hernia ring is well defined defect in the transverse aponeurosis [3].

Factors leading to increase tension on the abdominal wall aponeurosis or increase intra-abdominal pressure, such as straining due to many causes like bladder outlet obstruction, chronic cough, obesity or multiple pregnancies are also believed to be risk factors for development of Spigelian hernia [4].

Because of masking the Spigelian hernia by abdominal fat and the aponeurosis of the external oblique this make the diagnosis difficult [5]. The fascial margin around the defect is sharp so this advisable for risk of strangulation and incarceration which is about 24 % and that advisable for surgery which is done mostly traditionally by using an open approach with a primary suture repair, or by placing an onlay or sublay mesh [5].

This work has been reported in line with the SCARE criteria [6].

## 2 Case presentation

A 60 year old female, presented with a palpable lump at the right lower quadrant of the abdomen since 7 month before her presentation.

## 3 Clinical discussion

For the first time the swelling is small and painless then gradually increase in size and associated with dull aching pain. She denies any history of bowel or bladder alteration. No associated history of fever, nausea, vomiting, change in bowel habit or abdominal distension. The swelling appeared on walking, straining and subsides on lying down for first time but later on it was not but still reducible. Regarding other review of systems no significant symptoms. She was not smoker and not alcoholic. She had negative drug, medical and surgical history, also she had no psychological problem.

On clinical examination, vital parameters were within normal limits. On local examination, fullness was noted in right lower abdomen without signs of inflammation. On palpation she had a 6 cm × 6 cm well demarcated swelling in right iliac fossa lateral to rectus margin. It had a smooth surface and skin over the swelling had no scars sinuses or dilated veins. The swelling was reducible with a defect of size 4 × 4 cm palpable in right iliac fossa. There was a positive cough impulse. The swelling was non tender. Other hernial orifices were normal. No inguinal lymphadenopathy noted. Abdominal ultrasonography done revealed a defect in abdominal wall in right iliac fossa with reducible bowel content. Depending on basis of clinical and investigations, a diagnosis of Spigelian hernia was made. Her routine investigations were normal and her abdominal X-ray showed no signs of bowel obstruction.

**Fig. 1:**
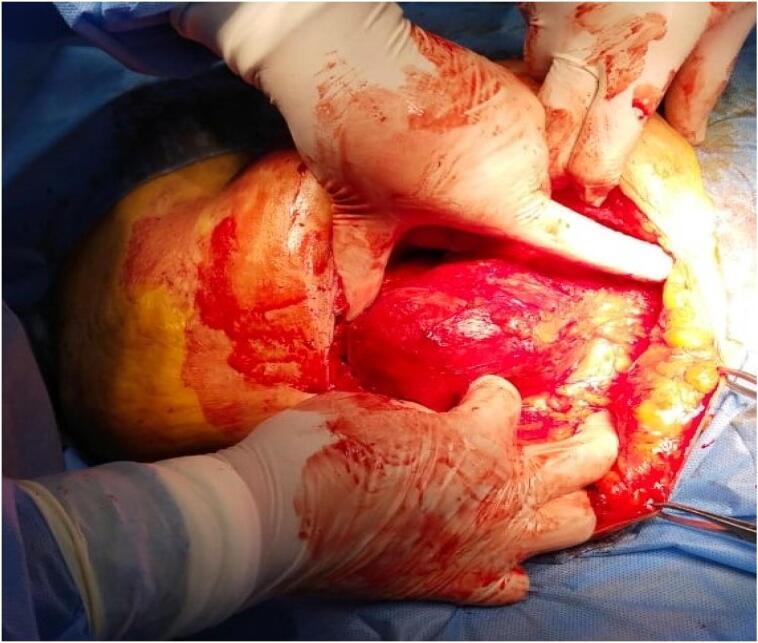
Herniation through a defect along the lateral border of rectus sheath.

After preparation for surgery, exploration done under general anesthesia by an oblique incision over the swelling and was found to have herniation through a defect along the lateral border of rectus sheath (Fig. 1).

**Fig. 2:**
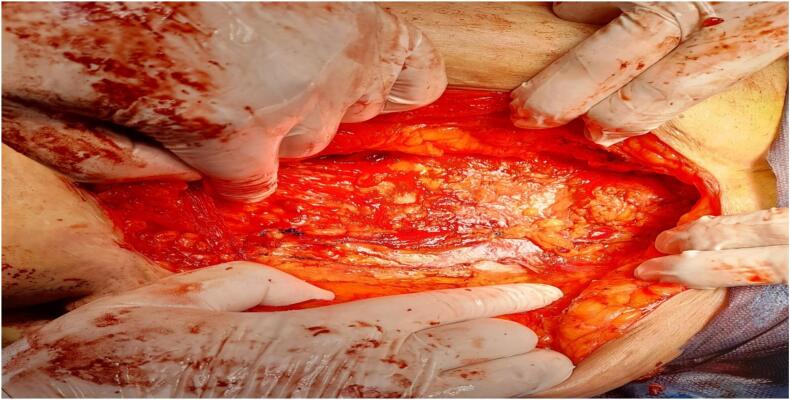
Anatomical repair of medial border of internal oblique and transverse abdominus muscle to the lateral border of rectum abdominal wall.

**Fig. 3:**
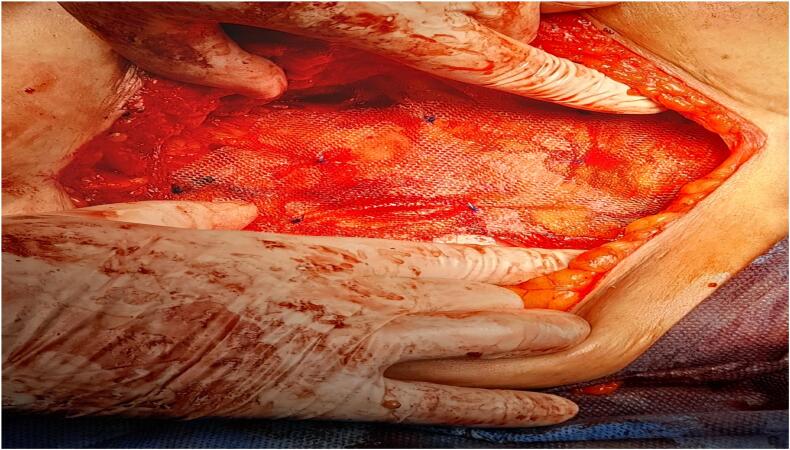
Hernioplasty by mesh.

The defect measuring 4 cm in length was identified and anatomical repair was done with nylon- 0, by suturing medial border of internal oblique and transverse abdominus muscle to the lateral border of rectum abdominal wall (Fig. 2) followed by hernioplasty by mesh (Fig. 3).

Post operatively, patient had an uneventful recovery. She followed up for 9 months after surgery where the patient was asymptomatic and clinical examination was unremarkable.

## 4 Discussion

Spigelian hernias are rare inter-parietal hernia which is also called “spontaneous lateral ventral hernia” or “hernia of semilunar line”. Sometimes it is called intraparietal, interstitial, intramuscular or intra mural hernia because it is locked between the different muscle layers of the abdominal wall [3]. The “Spigelian hernia belt” is the site where Spigelian hernia mostly occurs and it is located in a paramedian region lying 0 to 6 cm cranial to a line running between both anterior superior iliac spines [7]. The Spigelian fascia varies in width along the semilunar line and it gets wider as it approaches the umbilicus [3].

The hernia sac surrounded by extra peritoneal fatty tissue is often intra-parietal passing through transverse and internal oblique aponeurosis and spreading out behind the intact aponeurosis of external oblique [3]. The hernia sac usually contains the greater omentum but involvement of other organs has been reported, including the small intestine, colon, stomach, gallbladder, Meckel's diverticulum, appendix, ovaries and testes [7].

The preoperative diagnosis is often difficult because the clinical symptoms of Spigelian hernia are not characteristic due to the rarity of Spigelian defects and lack of personal clinical experience [8]. The diagnosis of Spigelian hernia based on history and physical examination alone depended in many cases but patients who underwent CT or US potentially provide information to aid in the proper diagnosis [7]. The deferential diagnosis of Spigelian hernia includes rectus sheath hematoma, abdominal wall abscess and seroma [8].

Always surgical intervention is the management of Spigelian hernias which is done either by open technique or laparoscopically, with a low recurrence rate after surgical repair, a lower morbidity and shorter hospital stay associated with laparoscopic technique [9]. In our patient, an open approach to the hernia allowed optimal exposure in a timely fashion.

## 5 Conclusion

Spigelian hernias are rare multifactorial disorder leading to defect in the transversus abdominis muscle in anterior abdominal wall and subsequent protrusion of visceral content through the hernia defect. Spigelian hernias carry a significant risk of incarceration and strangulation of its content. Clinical presentation is often vague, leading to delayed diagnosis. Physical examination along with CT scan is the most definitive radiologic test in establishing a diagnosis of a Spigelian hernia. Surgical intervention is the always the management of Spigelian hernias, which can be done in a traditional open fashion or laparoscopically and has a low recurrence rate after surgical repair.

## Consent

Written informed consent was obtained from the patient for publication of this case report and accompanying images. A copy of the written consent is available for review by the Editor-in-Chief of this journal on request.

## Ethical approval

Ethical approval for this study (Ethical Committee N° N/A) was provided by the Ethical Committee of Al-Kindy Teaching Hospital, Baghdad, Iraq on May 2022.

Protocol number: N/A.

## Funding

N/A.

## Author contribution

Dr. Safaa Hadi Abdulsattar Alshihmani

M.B.Ch.B - F.I.B.M.S

Al-kindy Teaching Hospital, Baghdad/Iraq

(Data collection, data analysis, writing the paper, completion the article)

## Guarantor

Safaa Hadi Abdulsattar Alshihmani.

## Research registration number

N/A.

## Conflict of interest statement

N/A.
